# Effect of Trace Metal Ions on the Conformational Stability of the Visual Photoreceptor Rhodopsin

**DOI:** 10.3390/ijms241311231

**Published:** 2023-07-07

**Authors:** Feifei Wang, Pol Fernandez-Gonzalez, Eva Ramon, Patricia Gomez-Gutierrez, Margarita Morillo, Pere Garriga

**Affiliations:** 1 Grup de Biotecnologia Molecular i Industrial, Centre de Biotecnologia Molecular, Departament d’Enginyeria Química, Universitat Politècnica de Catalunya-Barcelona Tech, Edifici Gaia, Rambla de Sant Nebridi 22, 08222 Terrassa, Catalonia, Spain; feifei.wang@upc.edu (F.W.); pol.fernandez.gonzalez@upc.edu (P.F.-G.); eva.ramon@upc.edu (E.R.); margarita.morillo@upc.edu (M.M.); 2 Departament d’Enginyeria Química, Universitat Politècnica de Catalunya-Barcelona Tech, Edifici ETSEIB, Av. Diagonal 647, 08028 Barcelona, Catalonia, Spain; patricia.gomez-gutierrez@upc.edu

**Keywords:** rhodopsin, trace metals, thermal stability, chromophore regeneration, photobleaching

## Abstract

Trace metals are essential elements that play key roles in a number of biochemical processes governing human visual physiology in health and disease. Several trace metals, such as zinc, have been shown to play important roles in the visual phototransduction process. In spite of this, there has been little research conducted on the direct effect of trace metal elements on the visual photoreceptor rhodopsin. In the current study, we have determined the effect of several metal ions, such as iron, copper, chromium, manganese, and nickel, on the conformational stability of rhodopsin. To this aim, we analyzed, by means of UV-visible and fluorescence spectroscopic methods, the effects of these trace elements on the thermal stability of dark rhodopsin, the stability of its active Metarhodopsin II conformation, and its chromophore regeneration. Our results show that copper prevented rhodopsin regeneration and slowed down the retinal release process after illumination. In turn, Fe^3+^, but not Fe^2+^, increased the thermal stability of the dark inactive conformation of rhodopsin, whereas copper ions markedly decreased it. These findings stress the important role of trace metals in retinal physiology at the photoreceptor level and may be useful for the development of novel therapeutic strategies to treat retinal disease.

## 1. Introduction

The vertebrate retina contains an array of cells with a photosensitive layer composed of rod and cone photoreceptor cells. Rod cells respond to dim light, whereas cone cells are strong light- and color-sensing cells that contain red, green, and blue cone opsin proteins [1,2,3,4,5]. Rhodopsin (Rho) is the photoreceptor protein of rod cells responsible for dim-light vision, and a prototypic model of class A G protein-coupled receptors (GPCRs). Rho was the first GPCR whose crystallographic three-dimensional structure was solved at atomic resolution [6,7]. Rho is made up of opsin—with a distinctive seven transmembrane helical architecture—and the 11-*cis*-retinal (11CR) chromophore covalently bound, through a protonated Schiff base (PSB) linkage to Lys296 at helix 7, in the transmembrane core of the receptor [8]. The first step in the visual phototransduction process is the capture of photons using the 11CR chromophore. Upon illumination, 11CR isomerizes to its all-*trans*-retinal (ATR) configuration instantaneously, and this causes a conformational change that leads to the formation of the active Metarhodopsin II (Meta II) species. The active Meta II state is formed through a complex cycle of short-lived photointermediates, including bathorhodopsin, lumirhodopsin, and Meta I, which are formed on the picosecond to millisecond time scale [9]. The Meta II conformation eventually decays, with the formed ATR subsequently leaving the retinal binding pocket [10,11]. The active Meta II conformation can activate the G protein transducin and elicit a cascade of biochemical reactions by means of downstream effectors that eventually result in a visual signal to the brain [12,13,14].

Trace metals are indispensable for biochemical processes and are important cofactors for up to 40% of proteins to function properly [15]. Furthermore, these trace elements play an essential role in human visual physiological function [16]. The absence or excess of trace elements may lead to various diseases [17,18]. For instance, insufficient iron levels can result in anemia, inadequate iodine intake can lead to thyroid disorders, and a deficiency in zinc can cause the dysfunction of retinal cells, contributing to the development of diverse eye diseases [19,20,21,22,23]. The role of different metal ions on retinal phototransduction has been poorly investigated, and the effect of these elements at the photoreceptor Rho level has not been analyzed to date. Therefore, it is important to clarify the potential effects of such ions at the molecular level on the photoreceptor protein Rho. This knowledge can shed light on the molecular mechanisms underlying the pathophysiology of visual disorders and facilitate the development of innovative therapeutic approaches to address them. The presence and accumulation of some metals in eye structures have been previously investigated, and they have been shown to be critical for visual function, especially at the retinal level [24,25]. In particular, changes in zinc levels have been linked to age-related eye diseases, vision loss, age-related macular degeneration, and cataracts [26,27]. Specific and nonspecific binding sites for zinc ions have been reported for Rho [28,29,30], and changes in these binding sites affect the stability of Rho. However, there are no available reports on the effects of other trace metal ions on the conformational stability of Rho, which is of interest to clarify the role of such metals at the photoreceptor cell structural level and particularly on the key photoreceptor protein Rho.

To study the effects of trace metal elements on Rho conformational properties, we have selected iron (Fe^3+^ and Fe^2+^), copper (Cu^2+^), chromium (Cr^3+^), manganese (Mn^2+^), and nickel (Ni^2+^), which are known to be present in the retina, as representative ions for our study. We have investigated the effect of such metals (in the form of chloride salts) on the chromophore thermal stability of the inactive (dark state) and active (Meta II formed after photoactivation) conformations and in the chromophore regeneration process of purified Rho. We find that Fe^3+^, but not Fe^2+^, clearly stabilizes the inactive dark state conformation of Rho, whereas Cu^2+^ destabilizes it. In turn, Cu^2+^ prevents chromophore regeneration and dramatically slows down the Meta II decay process.

## 2. Results

### 2.1. Purification and Spectroscopic Analysis of Rho Isolated from Rod Outer Segments (ROS) of Bovine Retinas

**Figure 1:**
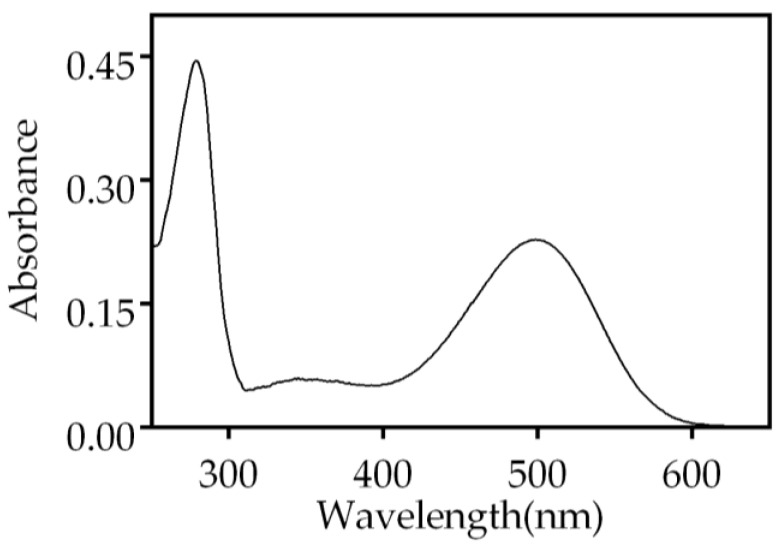
UV-vis absorption spectrum of immunopurified Rho from bovine ROS, in 2 mM sodium phosphate (NaPi), pH 6.0, and 0.05% n-dodecyl-β-d-maltoside (DM). The spectrum shows the characteristic bands at 280 nm (opsin) and 500 nm (11CR bound to opsin).

We purified bovine ROS Rho via immunochromatography using the Rho-1D4 monoclonal antibody and checked its purity via gel electrophoresis (Figure S1). Bovine Rho has been extensively used for structural studies because of its availability in large amounts. Therefore, it was of interest to investigate the effect of the metal ions on this Rho for a proper comparison with previously published data using purified Rho. The spectrum of the purified ROS Rho from bovine retinas showed the characteristic UV-vis profile with two main bands corresponding to opsin (280 nm) and 11CR covalently bound to opsin (500 nm) (Figure 1). The absorbance of Rho at 500 nm was 0.23, and the A_280_/A_500_ ratio was 1.95, which indicated a successful purification. This, and analogous samples, were used in the experiments described below.

### 2.2. Photobleaching and Acidification of Rho Treated with Different Trace Elements

**Figure 2:**
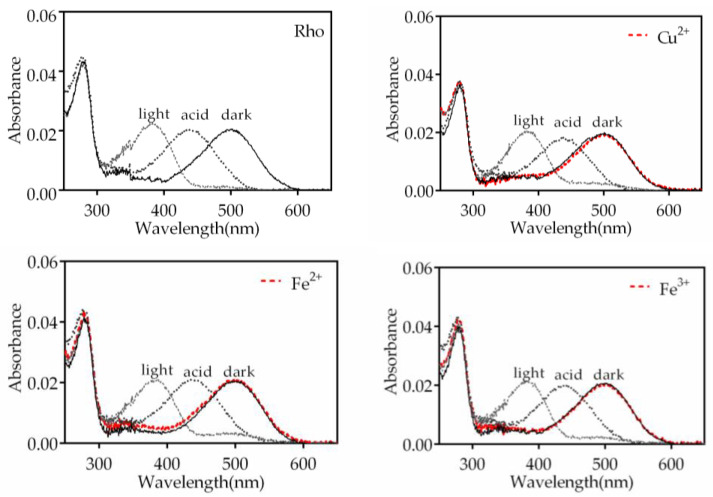
The UV-vis absorption spectra of Rho were obtained following pre-treatment with various metals under different experimental conditions. Spectra of samples were recorded in the dark state (dark, solid line), after metal addition (dark, dashed red line), upon photobleaching for 30 s (light, dashed line) and after acidification with 2N H_2_SO_4_ (acid, dotted line). Samples were, respectively, treated with Fe^3+^, Cu^2+^, and Fe^2+^ at a final concentration of 50 μM. All the above experiments were conducted at 20 °C.

Upon illumination, the PSB formed between 11CR and opsin undergoes deprotonation, resulting in a blue shift of the visible absorption band to 380 nm. Subsequently, the addition of H_2_SO_4_ causes the Schiff base (SB) to re-protonate, but the visual protein undergoes denaturation, leading to a red shift of the maximum absorption band to 440 nm. In this experiment, we treated the Rho samples with Fe^3+^, Fe^2+^, Cu^2+^, Cr^3+^, Mn^2+^, and Ni^2+^ individually and recorded the corresponding spectra (Figure 2 and Figure S2). The samples treated with Cr^3+^ and Mn^2+^ exhibited similar behavior to that of the control Rho sample without the addition of metal ion. However, samples treated with Fe^3+^, Fe^2^, and Cu^2+^ did not show complete conversion to the 380 nm species upon illumination, and we could detect some small remaining band at 500 nm. This may indicate the presence of some small fraction of PSB-linked species remaining in these cases.

### 2.3. Effects of Trace Metal Elements on Rho Thermal Stability in the Dark State

**Figure 3:**
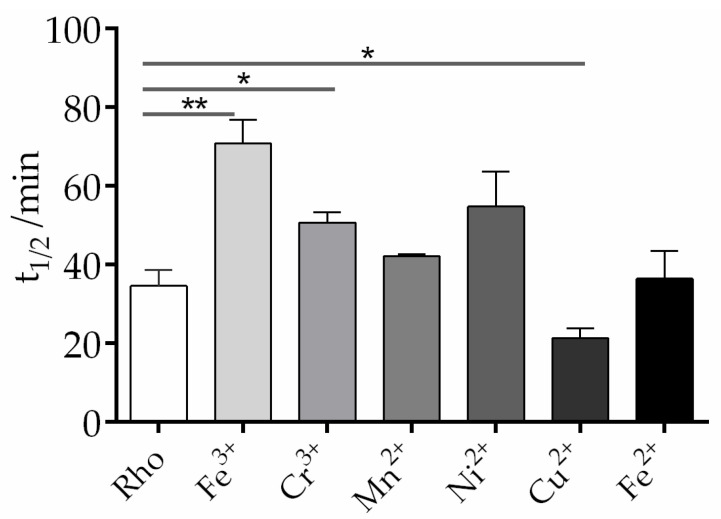
Effects of selected trace metals on the chromophore thermal stability of Rho in vitro. Under dark conditions, each trace metal was added separately to the sample at a final concentration of 50 μM. The absorbance of the sample was recorded in the wavelength range of 250 nm to 650 nm at 48 °C, with measurements taken every 2 min for a total duration of 100 min. The absorbance at 500 nm was plotted, and the t_1/2_ of the process was calculated based on the fitted curves. Mean and standard error of mean values were derived from independent repeated experiments (n = 3, * *p* < 0.05, ** *p* < 0.01).

Although the contents of trace elements in the human body is significantly low, they serve essential biological functions of utmost importance. These trace metals are involved in the metabolism of enzymes, hormones, and nucleic acids and also assist in the transport of macro elements. There are also different trace elements distributed in different areas of the eye, and among those we selected Fe^3+^, Cr^3+^, Mn^2+^, Ni^2+^, Cu^2+^, and Fe^2+^ to explore their effects on the chromophore thermal stability of the dark inactive conformation of Rho. The thermal decay process of Rho can be divided into two steps. In the first step, as the temperature increases, the 11CR bound to opsin undergoes isomerization to form ATR. In the second step, the deprotonated SB linkage is hydrolyzed and free ATR is released from opsin [31]. In our experiment, the chromophore thermal stability of the Rho samples, with and without metal elements, was tested using UV-Vis spectrophotometry in the dark at 48 °C. The thermal decay of purified Rho is clearly altered in different ways by the different metal ions (Figure 3). The half-life time (t_1/2_) for the process, particularly for Fe^3+^, but also for Cr^3+^, Ni^2+^, and, to a lesser extent, Mn^2+^, was clearly increased, indicating that these metal ions enhance the thermal stability of the dark inactive conformation of Rho. It is noteworthy that the other iron species, Fe^2+^, did not cause any change in the thermal stability compared to the control sample without any added metal ion. On the other side, Cu^2+^ produced a decrease in the thermal stability as judged by the lower t_1/2_ value of the corresponding decay process.

### 2.4. Effects of Trace Elements on the Chromophore Regeneration of Rho

**Figure 4:**
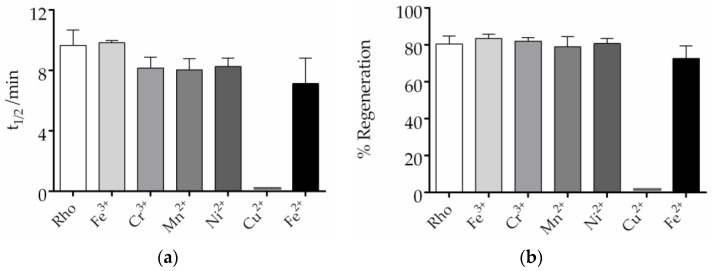
Effect of trace elements on the chromophore regeneration of Rho. The impact of trace elements on Rho regeneration was determined. Different metals and 11CR were added and the dark spectra were measured. Samples were subsequently photobleached, and spectra were recorded every 2 min at 20 °C min. The absorbance at 500 nm was plotted, and the mean and standard error of mean were calculated based on independent repeated experiments. (**a**) The t_1/2_ of the process was calculated from the fitted curves. (**b**) Regeneration percentage of Rho. The ratio of the amount of regenerated Rho to the original Rho was calculated. Mean and standard error of mean values were derived from independent repeated experiments (n = 3).

The regeneration of Rho is an important step in the visual process. The chromophore regeneration process was followed by measuring the increase at 500 nm after illumination of a Rho sample containing exogenously added free 11CR. The results obtained indicate that only Cu^2+^ affected the chromophore regeneration process by basically impairing it (Figure S3), but all the other metal ions did not significantly affect it (Figure 4). Analyses of the t_1/2_ of Rho regeneration showed no significant difference between the metal-treated and control untreated samples (Figure 4a). The t_1/2_ of the sample containing Fe^3+^ was essentially the same as that of the control sample, whereas the t_1/2_ of the other metal-containing samples was slightly lower than that of the control. Finally, the percentage of regenerated Rho, with respect to the original Rho, was determined, and it was found to be about 80% under our experimental conditions (Figure 4b). These results indicate that the metal ions, except for Cu^2+^, did not differentially affect the final percentage of chromophore regeneration.

### 2.5. Effects of Trace Elements on the Meta II Decay Process

Upon illumination, the 11CR covalently linked to opsin by means of a PSB linkage, changing its configuration to ATR. As a consequence, activated Meta II is formed which decays to free ATR and opsin with time. Under our experimental conditions, the Meta II decay process closely parallels the retinal release from the binding pocket. This allows an increase in Trp265 fluorescence to occur due to the fact that it was previously quenched by the presence of retinal in the binding pocket [9,32]. The effect of trace elements on Meta II can be analyzed by monitoring this fluorescence increase as a function of time.

**Figure 5:**
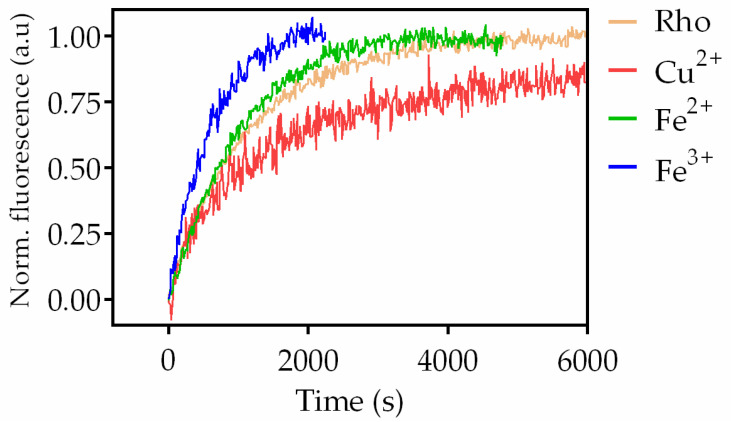
Meta II stability of Rho treated with or without metals. Fluorescence curves were recorded on a PTI Quanta Master 4 spectrofluorometer with a sample 0.5 µM Rho in the absence and the presence of metals at 20 °C. The fluorescence signal of Trp265 gradually increased over time, as a result of retinal leaving its binding pocket, until it reached a plateau.

**Figure 6:**
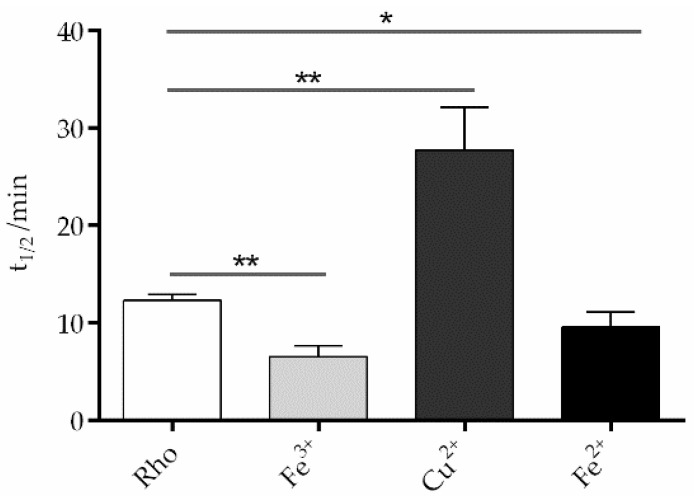
Effects of trace elements on Meta II t_1/2_. Upon excitation with light at 295 nm, Rho undergoes conformational changes, causing the release of bound retinal from the binding pocket and the fluorescence emission of a previously shielded Trp265. The fluorescence signal increase was recorded using a spectrofluorometer, and the t_1/2_ of the Meta II decay process was determined. Mean and standard error of mean values were derived from independent repeated experiments (n = 3, * *p* < 0.05, ** *p* < 0.01).

The fluorescence curves were recorded in the dark after illumination of the sample for 40 s at 20 °C (Figure 5). The samples treated with divalent iron and trivalent iron reached their maximum fluorescence faster than the control without metals and, consequently, had a reduced t_1/2_, particularly Fe^3+^ (Figure 6). However, the Cu^2+^-treated sample took much longer to reach its maximum fluorescence value, clearly reflecting a slower process compared to the other two cases. The behavior for chromium, manganese, and nickel samples was similar to that of the control sample (Figure S4). The sample treated with Cu^2+^ showed a much slower process than the control sample 27.7 ± 2.5 min vs. 12.3 ± 0.3 min, respectively. In the case of Fe^2+^ (9.6 ± 0.9 min) and Fe^3+^ (6.6 ± 0.6 min), these values reflected significantly faster processes compared to the control sample without metal ions. There was no significant difference in t_1/2_ between the control sample and the samples treated with chromium, manganese, and nickel, respectively.

### 2.6. Molecular Modeling of Fe^3+^ and Fe^2+^ Interaction with Rho

**Figure 7:**
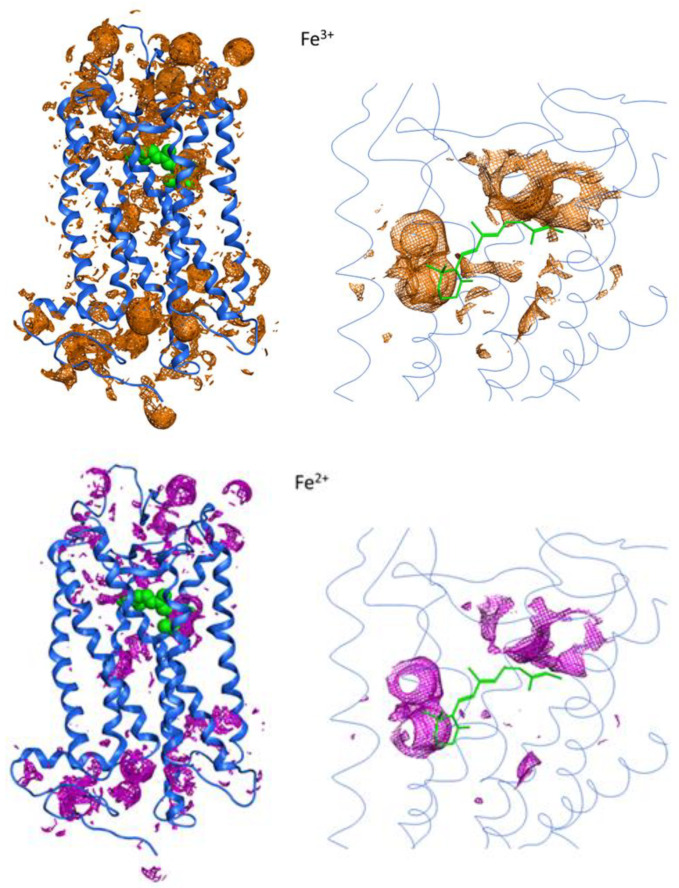
Fe^3+^ and Fe^2+^ binding sites on the surface of the crystal structure of bovine Rho (PDB ID: 1U19) determined by GRID22 program as implemented in MOE software (version MOE2020.09). Left models correspond to a full protein view, whereas the right models correspond to the magnified retinal binding pocket domain and are depicted in an inverted manner with regard to the left images for better visualization of the 11-*cis*-retinal chromophore. The secondary structure is represented in blue-colored ribbon, whereas retinal is shown in green using CPK (**left**) or stick representation (**right**). Surfaces in orange and purple are the calculated interaction potential surfaces for Fe^3+^ and Fe^2+^, respectively, using an iso-contour level of −12.5 kcal/mol.

Fe^3+^ and Fe^2+^ putative binding sites were identified by calculating the interaction potential maps. To this aim, Fe^3+^ and Fe^2+^ probes were passed through the crystal structure of bovine Rho. The results show that the interaction surfaces on Rho for Fe^3+^ are larger than those for Fe^2+^ at the same energy level (Figure 7). This indicates that Fe^3+^ has a higher affinity for the protein than Fe^2+^ and could explain the increased stability provided by Fe^3+^. These models may be reflecting electrostatic contributions to a significant extent, but more specific binding effects, such as those of a steric or structural nature, cannot be ruled out.

## 3. Discussion

Trace elements are indispensable cofactors for more than 40% of protein active sites and are very important for human health. Several studies have shown that the absence or deficiency of certain trace elements can lead to disease. Trace elements are found throughout the body, including the eyes, and their influence on retinal physiology in connection with visual health has been the matter of previous investigations [33]. In the case of zinc, there is a certain concentration of zinc in the retina, which appears to play an important role in the structural stability of the visual photoreceptor protein Rho. In this regard, abnormal concentrations of zinc in the retina can lead to vision loss and cataract formation [34]. In spite of this, there are scarce studies dealing with the effect of trace elements on Rho structure and conformational stability. To fill this gap, we have conducted an analysis of the effects of different selected trace metal ions, namely Fe^3+^, Cr^3+^, Mn^2+^, Ni^2+^, Cu^2+^, and Fe^2+^, on Rho conformation and structural stability.

We focused on the effect of trace elements on three main parameters of Rho: thermal stability, chromophore regeneration, and the decay of the active conformation of Rho, Meta II, formed upon illumination. A very interesting feature of our results is the differential effect of Fe^3+^ and Fe^2+^, particularly concerning the chromophore thermal stability in the dark state. These results indicate that only Fe^3+^ provides increased stability to Rho, but Fe^2+^ did not affect this property. Moreover, Fe^3+^ showed a more pronounced effect on the retinal release process than Fe^2+^. In contrast, the two iron species showed no significant effects on the chromophore regeneration process compared to the control sample (see Figure 4). This result provides increased evidence of the importance of metal ions homeostasis in biochemical processes and the relevance of this fine-tuned regulation. In this regard, the differential effect of iron in different oxidation states should be further stressed and taken into account in future studies. It has been recently reported that Fe^2+^, but not its Fe^3+^ counterpart, can cause oxidative stress and photoreceptor cell death in a mouse model of retinal degeneration [35]. Iron accumulation has also been associated with lipid peroxidation and ferroptosis as a result of the disturbance of iron homeostasis in age-related macular degeneration [36]. Fe^2+^ has also been associated with ferroptosis photoreceptor degeneration in mice with defects in ATR clearance [37].

On the other side, specific behavior of Cu^2+^ was also observed. In this case, this metal ion would cause an important decrease in thermal stability and at the same time would dramatically slow down the retinal release process, after photoactivation, and this would be a possible cause of its lack of chromophore regeneration. These results point to a deleterious effect of copper on the structural stability of retinal Rho, at least under our experimental conditions. A potential explanation of the observed behavior with Cu^2+^ may be found in the fact that a high binding affinity of copper for Rho was previously reported in a study where only this metal could compete for zinc binding to Rho [38]. This indicates that copper interacts with Rho and impairs retinal release and subsequent free 11CR uptake, and this would explain the lack of chromophore regeneration observed in the Cu^2+^-treated sample (Figure 4). Therefore, the significantly increased t_1/2_ value for copper (Figure 6) can be interpreted as a very slow retinal release process that would preclude chromophore regeneration by impairing free 11CR binding to the protein. In contrast, iron did show a faster process, indicating that retinal could readily enter the binding pocket to regenerate Rho. An alternative explanation for the effect seen for copper is that this metal ion would bind at a site which is in the pathway of retinal entry, and this would cause the observed lack of chromophore regeneration.

In conclusion, copper has a strong negative effect on Rho stability, possibly as a result of its specific binding to the photoreceptor protein, whereas Fe^3+^ is beneficial for improving Rho thermal stability. Interestingly, Fe^2+^ is not able to stabilize Rho towards thermal bleaching. A differential effect of iron at different oxidation states has been previously observed in other biochemical pathways [35,36]. Notably, the stabilizing effect of Fe^3+^, but not Fe^2+^, on Rho dark-adapted conformation detected in our study may have implications for retinal physiology and opens up novel avenues for the use of such metal in combination with other molecular entities in the development of successful therapeutic strategies to treat inherited visual disorders.

## 4. Materials and Methods

### 4.1. Materials

All metal compounds were used as chloride salts and were purchased from Sigma (Madrid, Spain). DM was purchased from Anatrace Inc. (Maumee, OH, USA). Bovine retinas were purchased from WL Lawson (Omaha, NE, USA). 11CR was provided by the National Eye Institute, National Institutes of Health (Bethesda, MD, USA), and ATR from Sigma (Madrid, Spain). The mAb rho-1D4 antibody was obtained from Cell Essentials (Boston, MA, USA). H-TETSQVAPA-OH (9-mer) peptide was synthesized by Unitat de Tècniques Separatives i Síntesi de Pèptids (Barcelona, Spain).

### 4.2. Methods

#### 4.2.1. Purification of Rho from ROS of Bovine Retinas

The whole purification process of Rho was carried out in the dark or under dim-red light of Kodak safelight filter-1521624. ROS membranes from bovine retinas were resuspended with 2 mM NaPi buffer, pH 6.0, and solubilized using 1% DM (*w*/*v*) by gently shaking for 1 h, and the samples were subsequently centrifuged. The supernatant was collected and mixed with 1D4-coupled Sepharose beads, gently nutated for 3 h, and centrifuged again. The beads were washed multiple times (at least 3 times), and Rho was eluted with a buffer containing the 9-mer peptide corresponding to the last 9 amino acids of the C-terminal tail of Rho. The absorbance of all samples was recorded with a Varian Cary 100 UV-Vis spectrophotometer in the dark at 20 °C. The concentration of the purified Rho was determined by measuring the absorbance at 500 nm with an ε = 40,600 M^−1^·cm^−1^.

#### 4.2.2. Photobleaching and Acidification

First, the spectrum of the sample in the dark state was recorded (dark spectrum). Then, the sample was exposed for 30 s to a Dolan-Jenner MI-150 light source (Boxborough, MA, USA), with a cut-off filter at 495 nm, and the spectrum after photolysis was recorded (light spectrum). Finally, 2N H_2_SO_4_ was added to the sample, and the spectrum was immediately recorded (acid spectrum). The maximum absorption peak of the sample exhibited a shift from 500 nm to 380 nm upon photobleaching, and to 440 nm upon subsequent acidification.

#### 4.2.3. Thermal Decay Kinetics in the Dark State

Thermal decay kinetics experiments were conducted with purified Rho in 2 mM NaPi buffer, pH 6.0, and 0.05% DM. First, the UV-vis spectrum in the 250–650 nm interval region of the Rho sample was measured in the dark at 20 °C. Then, the temperature of the instrument was set at 48 °C, and spectral cycles were measured to follow the decay of the visible absorption band with time. Samples with metals contained a final concentration of 50 µM (added from a concentrated stock of the corresponding chloride salt), and spectra of the corresponding samples without added metal were also measured as a control. The specific spectra acquisition parameters were 50 cycles in total, 2 min for each cycle, and a scan speed of 400 nm/min. Finally, the equation A_500_ = A/A_0_, where A is the absorbance recorded at 500 nm at different times and A_0_ is the original absorbance at 500 nm, was used to normalize the data, and the obtained curves were fitted to an exponential function. All experiments were repeated three times for statistical significance.

#### 4.2.4. Chromophore Regeneration Assay

The chromophore regeneration assay was carried out at 20 °C in the dark. Briefly, the initial dark UV-vis spectra of the Rho samples, with or without metal ions (final concentration 50 µM), were measured. Next, exogenous 11CR (from a concentrated stock ethanol solution) was added to the Rho sample, and the UV-vis spectrum was recorded again. Then, the Rho samples were bleached by means of a Dolan Jenner FIBER-LITE-MI-150 light source equipped with a λ > 495 nm cut-off filter for 40 s, and the increasing absorbance at the visible maximum was continuously measured with time. The spectral absorption data were recorded using the following procedure: the specific parameters were 50 cycles in total, 2 min for each cycle, and a scan speed of 400 nm/min. All experiments were repeated three times. The obtained data were finally curve-fitted to an exponential function and the t_1/2_ was derived.

#### 4.2.5. Meta II Decay by Fluorescence Spectroscopy

This assay was conducted on a Photon Technologies International Quanta Master 4 Spectrofluorometer (Birmingham, NJ, USA). A 0.5 µM Rho sample was added to a fluorometric cuvette. The whole experiment was carried out at 20 °C in the dark. The excitation wavelength was set to 295 nm and the sample was irradiated through a 0.5 nm beam slit for 2 s. Then, a beam shutter was used to block the excitation light for 28 s. The tryptophan fluorescence signal was recorded at 330 nm through a 10 nm slit. Initially, the sample was measured in the fluorometer, in the dark, until the fluorescence signal was stable, and the sample was then irradiated with a 150 W Dolan-Jenner Mi-150 power source using a λ > 495 nm cut-off filter for 40 s. The fluorescence signal of Trp265 gradually increased over time, as a result of retinal leaving its binding pocket, until it reached a plateau. Finally, the experimental data were fitted to an exponential function and the t_1/2_ of the process was determined.

#### 4.2.6. Molecular Modeling

Fe^3+^ and Fe^2+^ putative binding sites were identified by calculating the interaction potential maps using the GRID22 (Molecular Discovery Ltd., Borehamwood, UK) probes as implemented in the MOE software (version MOE2020.09). Accordingly, Fe^3+^ and Fe^2+^ probes were passed through the crystal structure of bovine rhodopsin (PDB ID: 1U19). Surfaces depicted in the model correspond to the calculated interaction potential surfaces for Fe^3+^ and Fe^2+^, respectively, using an iso-contour level of −12.5 kcal/mol.

#### 4.2.7. Statistical Analysis

The results were presented as the mean value ± standard error of the mean which was calculated from independent replicates (n = 3). Statistical analysis was performed using GraphPad Prism 6 (GraphPad Software Inc., San Diego, CA, USA). To determine the statistical significance of the findings, an unpaired two-tailed t-test was conducted with a significance level set at *p* < 0.05.

## Data Availability

All original spectral data used to obtain the spectral graphs are available upon request.

## Supplementary Materials

The supporting information can be downloaded at: https://www.mdpi.com/article/10.3390/ijms241311231/s1.
