# Supplementary material for: Effect of Trace Metal Ions on the Conformational Stability of the Visual Photoreceptor Rhodopsin
Source: Int J Mol Sci. 2023 Jul 7;24(13):11231. doi: 10.3390/ijms241311231 (PMC10342768; doi:10.3390/ijms241311231)
Supplement: Supplementary file 1 [file ijms-24-11231-s001.zip › ijms-2470112-supplementary.pdf]

Supplementary material

Effect of Trace Metal Ions on the Conformational Stability of the Visual Photoreceptor Rhodopsin

Feifei Wang, Pol Fernandez-Gonzalez, Eva Ramon, Patricia Gomez-Gutierrez, Margarita Morillo and Pere Garriga *

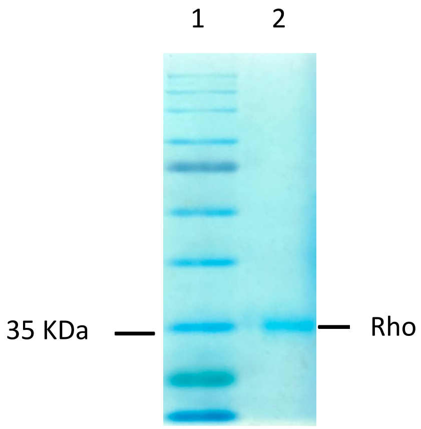

Figure. S1. SDS-PAGE electrophoresis of the Rho purified sample used in the characterization experiments. 1, MW marker; 2, Rho.

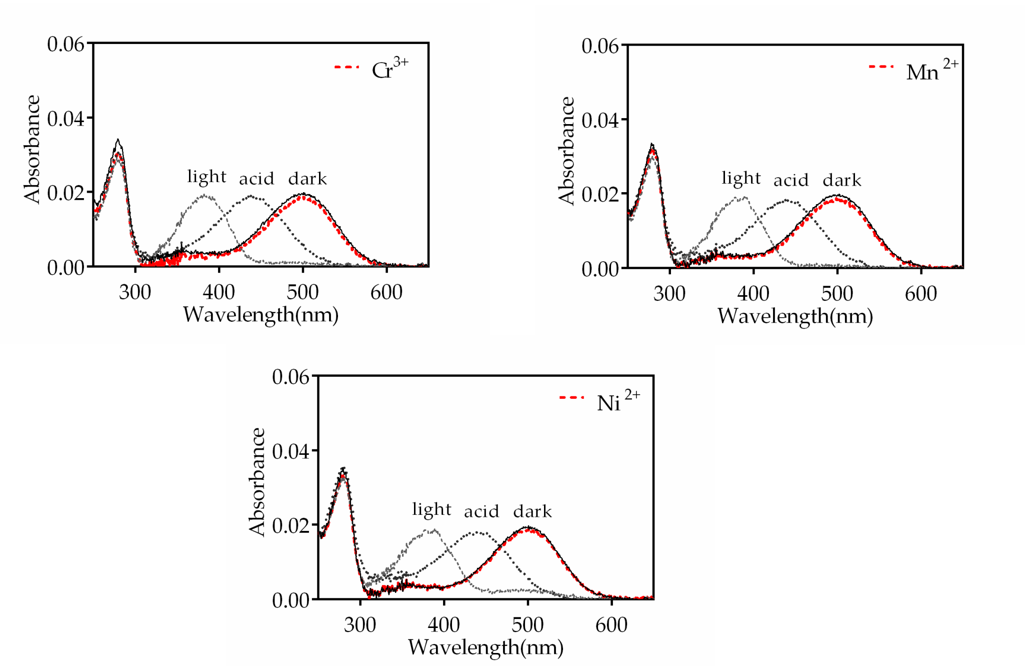

Figure S2. The UV-vis absorption spectra of Rho were obtained following pre-treatment with Cr^{3+}, Mn^{2+}, and Ni^{2+}, and final concentration is 50 μM. Spectra of samples were recorded in the dark state (dark, solid line), after metal addition (dark, dashed red line), upon photobleaching for 30 s (light, dashed line) and after acidification with 2N H_2SO_4 (acid, dotted line). All the above experiments were conducted at 20 °C.

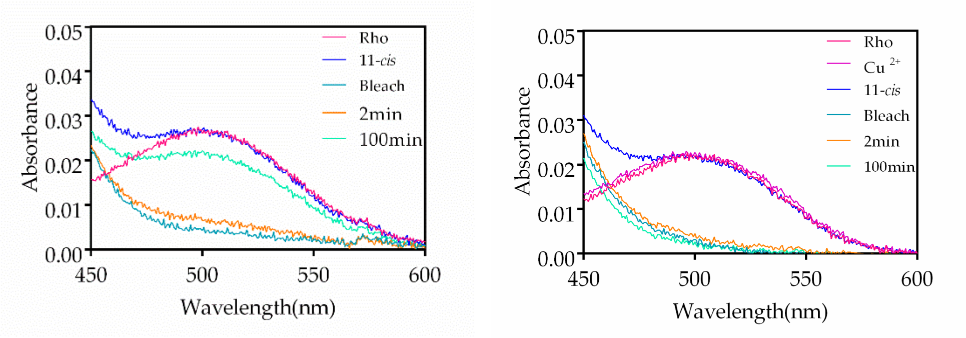

Figure S3. The UV-vis absorption spectra of regeneration (**left**) without and (**right**) with Cu^{2+}. The chromophore regeneration assay was carried out at 20 °C in the dark. The details are described in the Methods. 11-*cis* means adding 11-*cis* solution. Bleach means use light source to bleach sample. 2 min and 100 min means regeneration at 2 and 100min. From this figure, it indicated that with Cu^{2+} almost regenerated nothing.

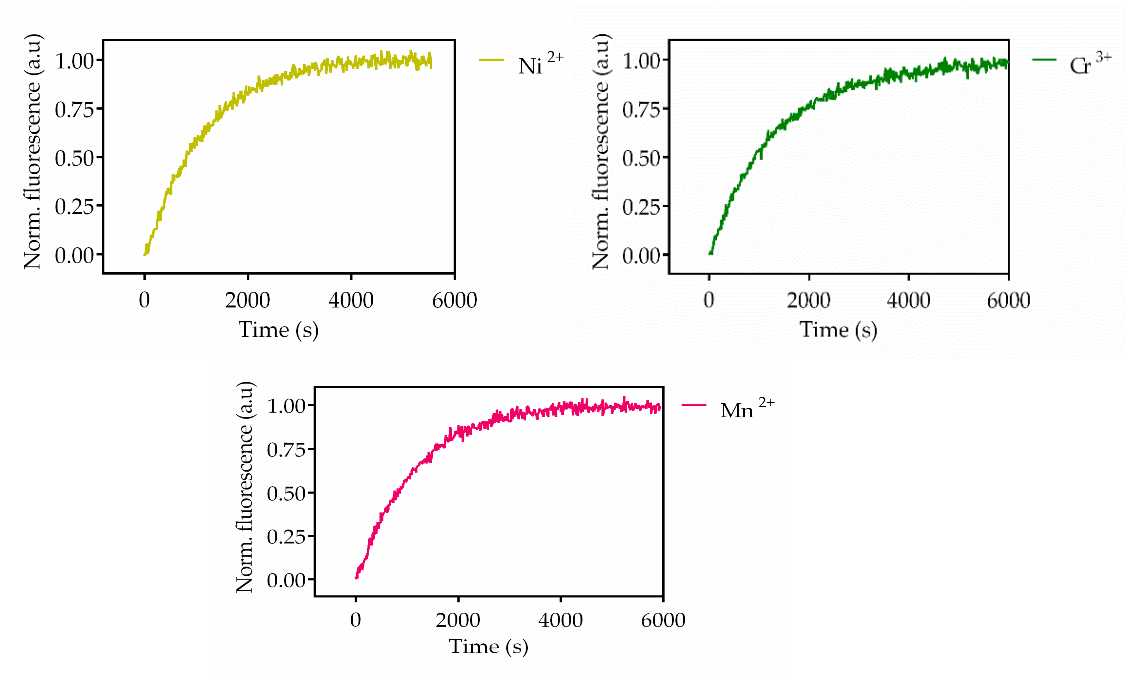

Figure S4. Meta II stability of Rho treated with Ni^{2+}, Cr^{3+} and Mn^{2+}. Fluorescence curves were recorded on a PTI Quanta Master 4 spectrofluorometer with a sample of 0.5 μM Rho in the absence and the presence of metals at 20 °C. The fluorescence signal of Trp265 gradually increased over time, as a result of retinal leaving its binding pocket, until it reached a plateau.
